# Immune response of heterologous versus homologous prime-boost regimens with adenoviral vectored and mRNA COVID-19 vaccines in immunocompromised patients

**DOI:** 10.3389/fimmu.2023.1187880

**Published:** 2023-06-12

**Authors:** Chang Chu, Anne Schönbrunn, Dorothea Fischer, Yvonne Liu, Johann-Georg Hocher, Jutta Weinerth, Kristin Klemm, Volker von Baehr, Bernhard K. Krämer, Saban Elitok, Berthold Hocher

**Affiliations:** ^1^ Fifth Department of Medicine (Nephrology/Endocrinology/Rheumatology/Pneumology), University Medical Centre Mannheim, University of Heidelberg, Mannheim, Germany; ^2^ Institute of Medical Diagnostics, Institute of Medical Diagnostics (IMD) Berlin-Potsdam, Berlin, Germany; ^3^ Department of Obstetrics, Ernst Von Bergmann Hospital Potsdam, Potsdam, Germany; ^4^ Charité - Universitätsmedizin Berlin, Berlin, Germany; ^5^ Department of Gastroenterology, Infectiology and Rheumatology, Ernst Von Bergmann Hospital Potsdam, Potsdam, Germany; ^6^ Department of Nephrology and Endocrinology, Ernst Von Bergmann Hospital Potsdam, Potsdam, Germany; ^7^ European Center for Angioscience ECAS, Faculty of Medicine of the University of Heidelberg, Mannheim, Germany; ^8^ Center for Preventive Medicine and Digital Health Baden-Württemberg (CPDBW), Medical Faculty Mannheim, Heidelberg University, Mannheim, Germany; ^9^ Mannheim Institute for Innate Immunoscience, Medical Faculty Mannheim of the University of Heidelberg, Mannheim, Germany; ^10^ Reproductive and Genetic Hospital of China International Trust Investment Corporation (CITIC)-Xiangya, Changsha, China

**Keywords:** heterologous vaccination, homologous vaccination, AstraZeneca adenoviral ChAdOx1-S-nCoV-19 vaccine, BioNTech mRNA BNT162b2 vaccine, immune response

## Abstract

Due to rare but major adverse reactions to the AstraZeneca adenoviral ChAdOx1-S-nCoV-19 vaccine (ChAd), German health authorities recommended adults under 60 who received one dose of ChAd, to receive a second dose of the BioNTech mRNA BNT162b2 vaccine (BNT) as a booster. Studies in the general population suggest an enhanced efficacy of the heterologous (ChAd-BNT) compared to the homologous (BNT-BNT) vaccination regimen. However, an analysis of the efficacy in patient populations with a high risk of severe COVID-19 due to acquired immunodeficiency is still missing. We therefore compared both vaccination regimens in healthy controls, patients with gynecological tumors after chemotherapy, patients on dialysis and patients with rheumatic diseases concerning the humoral and cellular immune response. The humoral and cellular immune response differed substantially in healthy controls compared to patients with acquired immunodeficiency. Overall, the most significant differences between the two immunization regimens were found in neutralizing antibodies. These were always higher after a heterologous immunization. Healthy controls responded well to both vaccination regimens. However, the formation of neutralizing antibodies was more pronounced after a heterologous immunization. Dialysis patients, on the other hand, only developed an adequate humoral and particularly cellular immune response after a heterologous immunization. Tumor and rheumatic patients also - to a weaker extent compared to dialysis patients - benefited from a heterologous immunization. In conclusion, the heterologous COVID-19 vaccination regimens (ChAd-BNT) seem to have an advantage over the homologous vaccination regimens, especially in immunocompromised patients such as patients with end-stage kidney disease treated with hemodialysis.

## Introduction

SARS-CoV-2 has a tremendous impact worldwide due to its high transmission and mortality rates. The disease spread rapidly despite strict policies such as the lockdown, and numerous people suffered from severe symptoms or even faced death. Vaccination was believed to be the game-changer of this pandemic (1, 2). Therefore, several types of vaccines were rapidly developed and urgently authorized at the end of 2020, including the two most widely used types: the mRNA vaccine Comirnaty (BNT162b2, Pfizer-BioNTech, BNT) and the adenoviral vectored vaccine Vaxzevria (ChAdOx1 nCoV-19, AstraZeneca, ChAd). Both were initially approved with a two-doses homologous vaccination regimen in Germany.

In March 2021, some European governments suspended the use of AstraZeneca’s ChAd in men and women under 60 due to safety concerns. This consequently led to a unique situation, where a mRNA-based heterologous booster was used despite the lack of further information on this heterologous regimen at the time (3). In addition, the shortage of SARS-CoV-2 vaccines made heterologous vaccinations an alternative to speed up the global rollout of the vaccinations, particularly in low- and middle-income countries. Given its widespread usage, concerns of safety, immunogenicity, and reactogenicity of heterologous prime-boost vaccinations were raised.

So far, a series of studies showed that a heterologous vaccine regimen (ChAd-BNT) is non-inferior to a homologous regimen (BNT-BNT) in terms of immunogenicity and prevention in healthy populations (4–8). However, the comparison of immunogenicity between the ChAd-BNT and the BNT-BNT vaccination has been far less conducted in immunosuppressed patients, such as patients with end-stage renal diseases, rheumatic patients, or oncology, which may affect immune responses. Currently, only in an observational study, levels of SARS-CoV-2-spike IgG were found to be significantly higher in hemodialysis patients with the ChAd-BNT vaccine regimen than with the BNT-BNT or ChAd-ChAd vaccine regimen (9). Therefore, we sought to compare the humoral and cellular immune response between the heterologous ChAd-BNT regimen and the homologous BNT-BNT regimen particularly in dialysis patients, gynecological oncology patients, and rheumatic patients from two independent hospitals (the Ernst von Bergmann Hospital and the University Medical Centre Mannheim) in Germany.

## Methods

### Study population

A total of 170 participants were enrolled, including 56 healthcare workers as healthy controls, 40 dialysis patients, 27 gynecological oncology patients, and 47 rheumatic patients, from two independent hospitals, the Ernst von Bergmann Hospital (Potsdam, Germany), and the University Medical Centre Mannheim (Mannheim, Germany). During the observation period (December 2020 to July 2021), participants were not infected with SARS CoV-2 and were fully vaccinated, either with homologous BNT vaccinations with a 3-week interval between vaccinations or heterologous ChAd-BNT vaccinations with a 12-week interval between vaccinations. Blood was drawn from these subjects at a median of seven weeks after the vaccinations. Examinations of all participants were conducted by study physicians with documentation of age, sex, body mass index (BMI), smoking status, comorbidities, i.e., type 1 or 2 diabetes, hypertension, chronic obstructive pulmonary disease (COPD), and asthma. The time from completion of vaccinations to blood collection was also recorded. The study was approved by the local ethics committee of the association of physicians. Written and informed consent was obtained from all participants in the study.

### Assessment of humoral responses to homologous (BNT-BNT) or heterologous (ChAd-BNT) COVID-19 vaccination regimens

The humoral immune response was assessed by IgG against SARS-CoV-2 spike glycoprotein 1 (S1), and the SARS-CoV-2 surrogate neutralization test. Serum anti-SARS-CoV-2 IgG (S1) was measured using IgG against SARS-CoV-2 spike glycoprotein 1 (S1) enzyme-linked immunosorbent assay (ELISA; EUROIMMUN) on an automated ANALYZER system (QuantiVac, EUROIMMUN) according to the manufactural instructions as previously described (10). Serum anti-SARS-CoV-2 IgG (S1) values above 35.2 BAU/ml were considered positive (10, 11). The SARS-CoV-2 surrogate neutralization test was assessed using a SARS-CoV-2 sVNT Kit (cPAss from Genscript) as previously described (10). Neutralizing antibody levels above 30% were considered positive (10, 11).

### Assessment of cellular responses to homologous (BNT-BNT) or heterologous (ChAd-BNT) COVID-19 vaccination regimens

Assessment of cellular responses was done by SARS-CoV-2 lymphocyte transformation test (SARS-CoV-2 LTT) as we published before (10, 11). Briefly, peripheral blood mononuclear cells (PBMCs) were isolated from heparinized venous blood by density gradient centrifugation and were resuspended in cell culture medium (RPMI 1640; Biowest) supplemented with 2 mM L-glutamine, 100 μg/ml gentamicin (all from Biowest) and 5% autologous serum. Specific T cell reactions were assessed by a lymphocyte proliferation assay. Therefore, incubation of PBMCs (2 x 10^5^) were performed in peptide pool 1 or 2 of SARS-CoV-2 spike glycoprotein (PM-WCPV-S from JPT) at a concentration of 1 µg/ml each, along with 1 µg/ml of anti-CD28 Abs (clone CD28.2 from BD Biosciences). The two pools both contained 15 peptides, each overlapping by 11 amino acids, spanning the entire SARS-CoV-2 spike glycoprotein. Pool 1 (N-Term) covered the N-terminal portion containing the RBD region and pool 2 (C-Term) covered the C-terminal portion of the protein (12). Positive controls were performed twice by stimulating the cells with a mixture of recalled antigens containing tetanus, influenza and candida albicans (antigen control) as well as with pokeweed mitogen (mitogen control, PWM, lectin from Phytolacca Americana, MERCK/Sigma) (13, 14). All stimulations were performed in triplicates in a 96-well plate at 37°C and 5% CO_2_ atmosphere for 5 days. The cells were labeled with 3H-thymidine (1 μCi/ml, Hartmann Analytic) 12 hours prior to cell harvest allowing for the tracking. Then a solid-phase β-counter (PerkinElmer) was used to determine the 3H-thymidine activity in counts per minute (cpm). The mean of the triplicates was calculated, and the results for each stimulus were finally given as a stimulation index (SI; ratio of cpm in cell culture with and without stimulation). The threshold SI for positivity was set at 1.9 (an SI >1.9 was considered positive) based on prior evidence (10, 11).

### Statistical analysis

Descriptive variables are shown as median values (interquartile ranges, IQR) or frequencies (percentages). Comparisons were performed using the Mann-Whitney U test or the Chi-Square (χ^2^) test, as appropriate. All statistical analyses were conducted using SPSS version 25.0 (SPSS, Chicago, IL, USA). The level of significance was set at p<0.05.

## Results

The cohort of this study comprises 56 healthcare workers, 40 dialysis patients, 27 gynecological oncology patients, and 47 rheumatic patients. Out of the patients with gynecological cancers, there were 21 with breast cancer, 5 with ovarian cancer, and 1 with endometrial cancer. All gynecological cancer patients received chemotherapy with or without monoclonal antibodies, except for three breast cancer patients who only received monoclonal antibodies. Of the rheumatic patients, there were 15 with rheumatoid arthritis, 13 with vasculitis, 5 with systemic lupus erythematosus, 2 with Sjogren’s syndrome, 2 with CREST syndrome, 2 with progressive systemic sclerosis, 2 with collagen vascular disease, 2 with psoriatic arthritis, 2 with spondyloarthropathy, 1 with pyoderma, and 1 with sarcoidosis. All rheumatic patients were treated with corticosteroids and/or conventional disease-modifying antirheumatic drugs (DMARDs) and/or biological DMARDs according to guidelines.

Among all participants, 32 healthcare workers, 19 dialysis patients, 23 gynecological oncology patients, and 38 rheumatic patients received vaccinations in the homologous regimen (BNT-BNT), while the remainder, 24 healthcare workers, 21 dialysis patients, 4 gynecological oncology patients, and 9 rheumatic patients, received heterologous vaccinations with a ChAd prime followed by a BNT boost (ChAd-BNT) (**Supplementary Figure 1**). On average, the healthcare workers were younger, had a lower BMI, and had a more robust immune response to the COVID-19 vaccine than patients with underlying diseases (**Table 1**).

**Table 1:** Characteristics of participants (healthcare workers, dialysis patients, gynecological oncology patients, and rheumatic patients).

Parameters	Healthcare workers (n=56)	Dialysis patients (n=40)	Gynecological Oncology patients (n=27)	Rheumatic patients (n=47)
Age (years)	41.0 (32.0, 51.3)	67.0 (62.3, 78.8) ^***^	54.0 (47.0, 76.0) ^###^	63.0 (47.0, 71.0) ^$$$^
Gender (M/F)	20/36	25/15^**^	0/27^###^	15/32
BMI	23.7 (21.3, 26.3)	26.0 (22.6, 29.9) ^*^	26.7 (23.9, 30.3) ^##^	25.4 (23.1, 31.2) ^$^
Diabetes (yes/no)	1/55	17/23^***^	2/25	9/38^$$^
Hypertension (yes/no)	6/50	36/4^***^	11/16 ^##^	21/26^$$$^
CHD	1/55	30/10^***^	1/26	4/43
COPD (yes/no)	1/55	10/30^***^	0/27	2/45
Asthma (yes/no)	7/48	1/39	4/23	2/45
Smoking (yes/no)	8/47	6/34	0/27^#^	6/41
SARS-CoV-2 IgG-Ab (S1) (BAU/ml)	768.0 (768.0, 768.0)	768.0 (84.5, 768.0) ^**^	148.0 (44.5, 768.0) ^###^	284.0 (83.3, 768.0) ^$$$^
SARS surrogate neutralization test (%)	96.0 (90.5, 97.0)	76.0 (14.3, 95.8) ^***^	59.0 (13.0, 97.0) ^###^	85.0 (42.0, 95.0) ^$$$^
Spike-N-Term LTT (SI)	6.4 (4.8, 10.9)	3.4 (1.3, 7.1) ^***^	2.8 (1.4, 4.9) ^###^	3.6 (1.6, 5.7) ^$$$^
Spike-C-Term LTT (SI)	4.8 (3.8, 8.5)	3.2 (1.3, 6.7) ^**^	2.8 (1.3, 6.4) ^##^	3.9 (1.4, 6.9) ^$$^

Variables are given as medians (interquartile range) or frequencies. Body mass index (BMI) was calculated as weight in kilograms divided by height in meters squared. CHD, coronary heart disease; COPD, chronic obstructive pulmonary disease. Comparisons were made using the nonparametric Mann-Whitney U test for continuous variables and the Chi-Square test for categorical variables. *p<0.05; **p<0.01; ***p<0.001, comparison between dialysis patients and healthcare workers; #p<0.05; ##p<0.01; ###p<0.001, comparison between gynecological oncology patients and healthcare workers; $p<0.05; $$p<0.01; $$$p<0.001, comparison between rheumatic patients and healthcare workers.

Immunosuppressed patients (with dialysis or gynecological oncology, or rheumatic patient) had significantly weaker immune responses in terms of both humoral and cellular immune response parameters compared to healthcare workers (p<0.0001 for SARS-CoV-2 IgG-Ab (S1), SARS-CoV-2 surrogate neutralization tests and Spike-N-Term LTT (SI), p=0.0003 for Spike-C-Term LTT (SI)). Further, this difference was more pronounced in immunosuppressed patients receiving the homologous regimen (BNT-BNT). The rates of positive immune responses were also lower in immunosuppressed patients than in healthcare workers and even lower in immunosuppressed patients with the homologous regimen (BNT-BNT) (**Table 2**).

**Table 2:** Humoral and cellular response to COVID-19 vaccine in immunocompromised patients (patients on hemodialysis, gynecological oncology patients, or rheumatic patients) and healthcare workers.

	SARS-CoV-2 IgG-Ab (S1) (BAU/ml)	SARS-CoV-2 IgG-Ab (S1) (P/N)	SARS surrogate neutralization test (%)	SARS surrogate neutralization test (P/N)	Spike-N-Term LTT (SI)	Spike-N-Term LTT (P/N)	Spike-C-Term LTT (SI)	Spike-C-Term LTT (P/N)
**Health care workers** (N=56)	768.0 (768.0, 768.0)	56/0	96.0 (90.5, 97.0)	55/1	6.4 (4.8, 10.9)	44/1	4.8 (3.8, 8.5)	44/1
*BNT-BNT (N=32)*	768.0 (376.3, 768.0)	32/0	93.0 (77.3, 96.0)	32/0	6.3 (4.1, 8.5)	20/1	5.4 (4.4, 9.2)	20/1
*ChAd-BNT (N=24)*	768.0 (768.0, 768.0)	24/0	97.0 (95.3, 97.0)	23/1	6.8 (5.2, 11.3)	24/0	4.6 (3.4, 6.8)	24/0
P value*	0.073	–	0.0003	0.429	0.255	0.467	0.179	0.467
**Immunocompromised patients** (N=114)	279.0 (70.5, 768.0)	97/17	79.0 (21.0, 95.0)	80/34	3.6 (1.4, 5.6)	73/41	3.3 (1.4, 6.7)	72/42
*BNT-BNT (N=80)*	182.5 (45.9, 768.0)	64/16	62.0 (14.0, 92.0)	48/32	2.7 (1.3, 5.4)	44/36	2.7 (1.3, 6.4)	44/36
*ChAd-BNT (N=34)*	768.0 (768.0, 768.0)	33/1	96.0 (82.0, 97.0)	32/2	4.6 (2.2, 8.2)	29/5	4.1 (2.1, 7.1)	28/6
P value*	<0.0001	0.021	<0.0001	0.0001	0.012	0.003	0.071	0.006

Variables are given as medians (interquartile range) or frequencies. Comparisons were made using the nonparametric Mann-Whitney U test or the Chi-Squared test for categorical variables. P, positive; N, negative. The threshold values of SARS-CoV-2 IgG-Ab (S1), SARS surrogate neutralization test, Spike-N-Term LTT, and Spike-N-Term LTT for positivity were set at above 35.2 BAU/ml, 30%, 1.9 SI, and 1.9 SI, respectively. P value*, comparison between the homologous regimen (BNT-BNT) and the heterologous regimen (ChAd-BNT).

**Figure 1:**
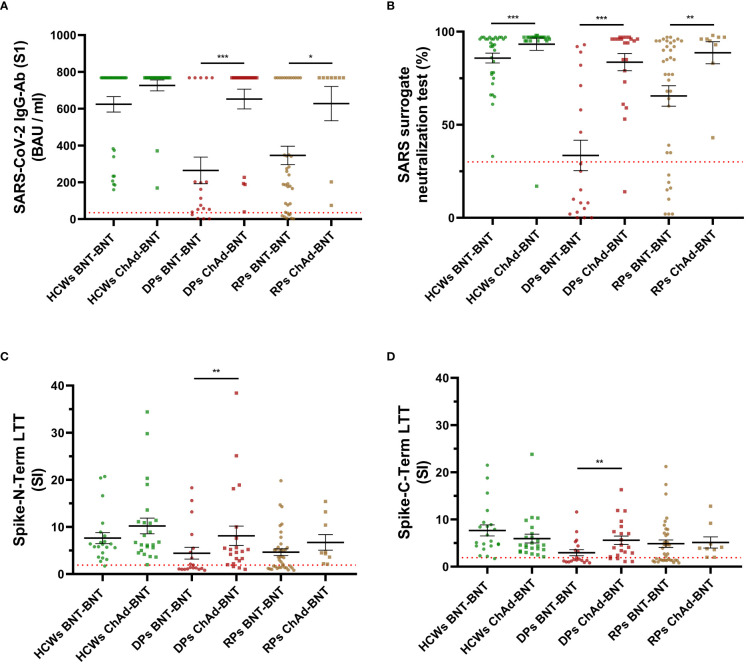
Scatterplots of humoral and cellular response to homologous (BNT-BNT) or heterologous (ChAd-BNT) COVID-19 vaccination regimens in participants with different medical conditions. All plots display geometric means with standard error of the mean (SEM). Statistical significance was assessed by Mann-Whitney U test. *, p<0.05, **, p<0.01, ***, p<0.001. HCWs, healthcare workers; ChAd, ChAdOx1-S-nCoV-19 vaccine; BNT, BioNTech mRNA BNT162b2 vaccine; DPs, dialysis patients; RPs, rheumatic patients; LTT, lymphocyte transformation test. **(A)** Scatterplots of SARS-CoV-2 IgG-Ab (S1) (BAU/ml). Red dotted line indicates the threshold value at 35.2 BAU/ml, n = 0, 0, 4, 0, 8, 0. **(B)** Scatterplots of SARS surrogate neutralization test (%). Red dotted line indicates the threshold value at 30%, n = 0, 1, 12, 1, 8, 0. **(C)** Scatterplots of Spike-N-Term LTT. Red dotted line indicates the threshold value at 1.9 SI, n = 1, 0, 11, 3, 15, 0. **(D)** Scatterplots of Spike-C-Term LTT. Red dotted line indicates the threshold value at 1.9 SI, n = 1, 0, 12, 4, 16, 0.

For the 56 healthcare workers, participants who received homologous BNT-BNT had a weaker humoral response shown in the SARS-CoV-2 surrogate neutralization test (%), compared to participants who received heterologous ChAd-BNT (p=0.0003, **Table 3**, **Figure 1**). On the other hand, regarding the positivity rate of both the SARS-CoV-2 IgG-Ab (S1) and the SARS-CoV-2 surrogate neutralization test, there was no statistical difference; the positive rate of humoral immunity was 100% in all participants, except for one participant that received the ChAd-BNT regiment, in the SARS-CoV-2 surrogate neutralization test (**Table 3**). However, in dialysis and rheumatic patients, both tests consistently showed a noticeably enhanced activation of the humoral immune system in heterologous ChAd-BNT receivers compared to homologous BNT-BNT receivers (**Table 3**, **Figure 1**). For dialysis patients, the positive rate of humoral immunity was significantly higher in participants receiving ChAd-BNT compared to BNT-BNT (100% vs. 78.9% for SARS-CoV-2 IgG-Ab (S1) and 95.2% vs. 36.8% for SARS-CoV-2 surrogate neutralization tests, respectively, **Table 3**). Positivity for SARS-CoV-2 IgG-Ab (S1) was 100% in patients with rheumatic diseases receiving ChAd-BNT compared to 78.9% in patients receiving BNT-BNT, and a similar trend was found in the SARS-CoV-2 surrogate neutralization tests (**Table 3**). In patients with gynecological cancer, only 4 patients received a heterologous regimen and thus limiting the ability to draw any conclusion, but both tests showed similar trends: the heterologous vaccination tends to stimulate a stronger humoral response. In dialysis patients, there were significant differences in cellular response between different vaccination regimens. The heterologous vaccination triggered a more potent cellular immune response (p=0.022 for Spike-N-Term LTT, p=0.008 for Spike-C-Term LTT, **Table 3**, **Figure 1**). In addition, the positive rate of cellular response was also significantly higher in the dialysis patients receiving ChAd-BNT compared to BNT-BNT (85.7% vs. 42.1% for Spike-N-Term LTT, p=0.007 and 81.0% vs. 36.8% for Spike-C-Term LTT, p=0.009, **Table 3**). This enhanced cellular response was not found in the rest of the study subgroups (**Table 3**, **Figure 1**). Nevertheless, the positive rates of both Spike-N-Term LTT and Spike-C-Term LTT were 100% in patients with rheumatic diseases receiving ChAd-BNT compared to 60.5% and 57.8% in patients receiving BNT-BNT (p=0.041 and p=0.019 respectively) (**Table 3**).

**Table 3:** Immune responses and positivity to COVID-19 vaccine in all participants (healthcare workers, dialysis patients, gynecological oncology patients, and rheumatic patients). .

Healthcare workers	SARS-CoV-2 IgG-Ab (S1) (BAU/ml)	SARS-CoV-2 IgG-Ab (S1) (P/N)	SARS surrogate neutralization test (%)	SARS surrogate neutralization test (P/N)	Spike-N-Term LTT (SI)	Spike-N-Term LTT (P/N)	Spike-C-Term LTT (SI)	Spike-C-Term LTT (P/N)
BNT-BNT (N=32)	768.0 (376.3, 768.0)	32/0	93.0 (77.3, 96.0)	32/0	6.3 (4.1, 8.5)	20/1	5.4 (4.4, 9.2)	20/1
ChAd-BNT (N=24)	768.0 (768.0, 768.0)	24/0	97.0 (95.3, 97.0)	23/1	6.8 (5.2, 11.3)	24/0	4.6 (3.4, 6.8)	24/0
P value	0.073	–	0.0003	0.429	0.255	0.467	0.179	0.467
Dialysis patients								
BNT-BNT (N=19)	113.0 (38.7, 768.0)	15/4	15.0 (3.0, 71.0)	7/12	1.4 (1.1, 5.5)	8/11	1.5 (1.2, 4.4)	7/12
ChAd-BNT (N=21)	768.0 (768.0, 768.0)	21/0	95.0 (76.0, 96.0)	20/1	4.8 (2.5, 8.8)	18/3	4.1 (2.3, 8.0)	17/4
P value	0.0003	0.042	<0.0001	0.0001	0.022	0.007	0.008	0.009
Rheumatic patients								
BNT-BNT (N=38)	249.5 (66.6, 768.0)	30/8	77.0 (35.0, 94.5)	30/8	3.6 (1.4, 5.6)	23/15	3.3 (1.4, 7.0)	22/16
ChAd-BNT (N=9)	768.0 (485.0, 768.0)	9/0	96.0 (88.0, 97.0)	9/0	4.5 (2.9, 11.8)	9/0	3.9 (2.8, 7.0)	9/0
P value	0.025	0.323	0.007	0.323	0.156	0.041	0.409	0.019

Variables are given as medians (interquartile ranges) or frequencies. Comparisons were made using the Mann-Whitney U test for quantitative variables and Chi-Square test for categorical variables. P, positive; N, negative. The threshold value of SARS-CoV-2 IgG-Ab (S1), SARS surrogate neutralization test, Spike-N-Term LTT, and Spike-N-Term LTT for positivity was set at above 35.2 BAU/ml, 30%, 1.9 SI, and 1.9 SI, respectively.

## Discussion

In our study, we conducted a head-to-head comparison of a heterologous (ChAd prime followed by BNT boost) with a homologous BNT-BNT vaccination regimen with regards to the humoral and cellular immune response in patient populations with different degrees of immune impairment. The humoral response - particularly seen in the neutralizing antibodies - was always more pronounced than the cellular response. Patients with end-stage renal disease on hemodialysis benefited most from the heterologous vaccination regimen by far. Homologous vaccination in dialysis patients results in a non-significant specific cellular and humoral immune response, resulting in inadequate protection against COVID-19. This pattern is fundamentally different after the heterologous combination of a ChAd prime followed by a BNT boost. Only after this heterologous regimen, there was an adequate humoral and cellular immune response in dialysis patients. Our study showed similar trends in patients with gynecological tumors and rheumatic diseases. Again, the heterologous immunization scheme led to a stronger immune response in these populations. On the other hand, in the healthy control population and to a lesser extent, the homologous vaccination regimen alone led to an adequate humoral and cellular immune response. But even in these populations, the response of neutralizing antibodies was more pronounced after heterologous vaccinations. It remains, however, questionable whether this slight difference in subjects with no or only minor impairment of the immune system is of medical significance.

Antibodies that bind to COVID-19 virus spike protein and prevent their entry into cells, referred to as neutralizing antibodies, proved crucial in the protection against COVID-19, and its level correlates with clinical protection (15). Once the virus enters the cell, T-cells play a pivotal role. Briefly, naive T-cells are activated and then turned into functional T-cells that either kill certain cells (cytotoxic T-cells) or modulate the immune response (helper T-cells) (16). Preclinical studies of vaccines development using heterologous combinations (adenoviral vectors and mRNA vaccines) showed strong immunogenicity (17). The cellular immune response following the heterologous regimen was dominated by CD8+ cytotoxic T-cells and Type 1 CD4+ helper T-cells (Th1), which was superior to the response induced by the homologous regimen in mice, thus suggesting the potential of heterologous administration (18). Studies in humans are in line with these preclinical findings, showing that in the general population with no or at most moderate impairment of the immune system, a heterologous ChAd-BNT vaccination scheme caused a more robust immunogenicity compared to homologous ChAd-ChAd or BNT-BNT vaccination combinations (5, 7, 19–21). Our data collected in healthy controls agree with the above-mentioned studies, showing a slightly enhanced immune response after administering the heterologous ChAd-BNT vaccination (5, 7, 8).

A key finding of our study was that both humoral and cellular immune responses after a heterologous ChAd-BNT vaccination regimen are superior to the responses induced by a homologous BNT-BNT vaccination regimen in dialysis patients. It is known that uremia is associated with immune dysfunction, characterized by the immunosuppression of the innate and adaptive immune system, which may lead to an increased rate of infection in these patients (16). Functional abnormalities of monocytes, neutrophils, and dendritic cells are directly linked to infection risk in this fragile population (16, 22, 23). In addition, high failure rates of certain vaccinations, such as vaccinations against hepatitis B virus, influenza virus, Clostridium tetani, or Corynebacterium diphtheriae, have also been reported in dialysis patients. They are considered to be caused by alterations in T-lymphocyte function (24). In the context of COVID-19, several studies have indicated that end-stage kidney disease patients on dialysis are not only particularly vulnerable to SARS-CoV-2 infections but are also at an increased risk of severe COVID-19 disease compared to patients without kidney failure (25, 26). This population’s impaired immunity and the high comorbidity rate resulted in a high overall mortality rate of 31% with COVID-19 (25, 26). Moreover, compared to healthy controls, dialysis patients have been documented to have a reduced antibody response (27). Altogether, these findings reinforce the importance of infection control measures for this vulnerable population. Findings from prior studies (4–8), together with our results, suggest an immunologic benefit of the heterologous ChAd-BNT regimen, the administration of heterologous ChAd-BNT is a promising and effective vaccination strategy. In our study, this benefit is even more pronounced in dialysis patients.

So far, underlying mechanisms of the immunological benefits of heterologous vaccinations remain largely unclear. Just like mRNA vaccines, adenovirus vector vaccines are designed to produce native S-proteins from a specific mRNA in cells of the vaccinee (28). However, the mRNA pathway in adenovirus vector vaccines is much more complex than in mRNA vaccines. It involves a bypass of the adenoviral DNA through the nucleus and requires specific additional cellular processes, including RNA transcription and processing (28). Besides, mRNA and adenovirus-vector vaccines elicit substantially different innate responses, which undoubtedly influence the nature of adaptive immune responses (29). After a single dose, the mRNA vaccines evoke just detectable non-neutralizing antibodies and moderate Th1-cell responses but almost no neutralizing antibodies. It has been reported that a booster immunization is required to reach detectable neutralizing antibody levels after immunization with SARS-CoV-2 mRNA vaccines (30), suggesting that the secondary antibody response may be derived mainly from memory B-cells produced by the first immunization. Contrastingly, adenovirus vaccines elicit polyfunctional antibodies even after a single dose, which can mediate viral neutralization, and drive other antibody-dependent effector functions and a robust T-cell response (31). Bruno Pozzetto et al. thus hypothesized that ChAd and BNT formulations lead to different memory B-cell compartments, and memory B-cells generated by ChAd elicitation may carry antigen receptors that show greater epitope recognition breadth or are more suitable for SARS-CoV-2 spike proteins. This may be related to the different conformations of the spike protein, as the BNT mRNA vaccine bears a mutation that stabilizes the protein in the pre-fusion conformation (7). In addition, it has been previously observed that mRNA vaccinations elicit extremely high neutralizing and conjugated antibody titers but relatively low CD8+ T-cell responses (31, 32). In contrast, adenovirus vector vaccines evoke lower levels of neutralizing and conjugating antibodies but cause the production of polyclonal antibodies after vaccination (33).

Vaccinations with different vaccine classes result in an enhanced vaccination response due to the parallel activation of different immunological mechanisms through different vaccine classes. This phenomenon is also consistent with previous studies reporting increased antibody responses in patients receiving a single dose of BNT and having already recovered from a natural SARS-CoV-2 infection, compared to seronegative individuals receiving two doses of BNT. These findings indicate that immune responses generated in different ways lead to more robust protection (5, 34, 35). A recent published study (7) suggested that an enhanced T-cell response after a heterologous COVID-19 vaccination might be particularly important for patients with a compromised immune system. This was exactly what was seen in the dialysis patients of our study – the subgroup that benefited most from a heterologous COVID-19 vaccination regimen. Our data also fit very well with a recently published study. This study showed a class switch towards non-inflammatory, spike-protein specific IgG4 antibodies after repeated SARS-CoV-2 mRNA vaccination leading potentially to a reduced neutralization capacity of the SARS Cov-2 virus. This phenomenon was not seen after heterologous vaccination (36).

Although our study showed clear findings, it also has certain limitations. The molecular mechanisms to explain this special benefit for dialysis patients are still unclear. The number of rheumatic and cancer patients receiving a heterologous COVID-19 vaccination regimen was lower compared to the other groups, thereby limiting findings in this subpopulation. It would also be interesting to study other populations with immunodeficiencies, for example patients with primary immunodeficiency diseases.

## Conclusions

The heterologous COVID-19 vaccination regimen seems to have a clear advantage over the homologous vaccination regimen, especially in immunocompromised patients such as patients with end-stage kidney disease on hemodialysis. Prospectively, controlled data regarding different COVID-19 vaccination regimens in different patient populations are needed.

## Data availability statement

The raw data supporting the conclusions of this article will be made available by the authors, without undue reservation.

## Ethics statement

The studies involving human participants were reviewed and approved by the Local Ethics Committee of the Association of Physicians. The patients/participants provided their written informed consent to participate in this study.

## Author contributions

BH designed the study. AS, DF, KK, and VvB processed samples and performed laboratory measurements. CC, J-GH, JW, and KK contributed to the data collection. CC and AS analyzed the results and composed all figures and tables. CC and BH drafted the manuscript. YL and J-GH contributed to professional language editing. BH, SE, and BKK contributed to the supervision of the analysis and revised the manuscript. All authors contributed to the article and approved the submitted version.
