# Supplementary material for: Immune response of heterologous versus homologous prime-boost regimens with adenoviral vectored and mRNA COVID-19 vaccines in immunocompromised patients
Source: Front Immunol. 2023 Jun 12;14:1187880. doi: 10.3389/fimmu.2023.1187880 (PMC10291065; doi:10.3389/fimmu.2023.1187880)
Supplement: Supplementary file 1 [file Image_1.pdf]

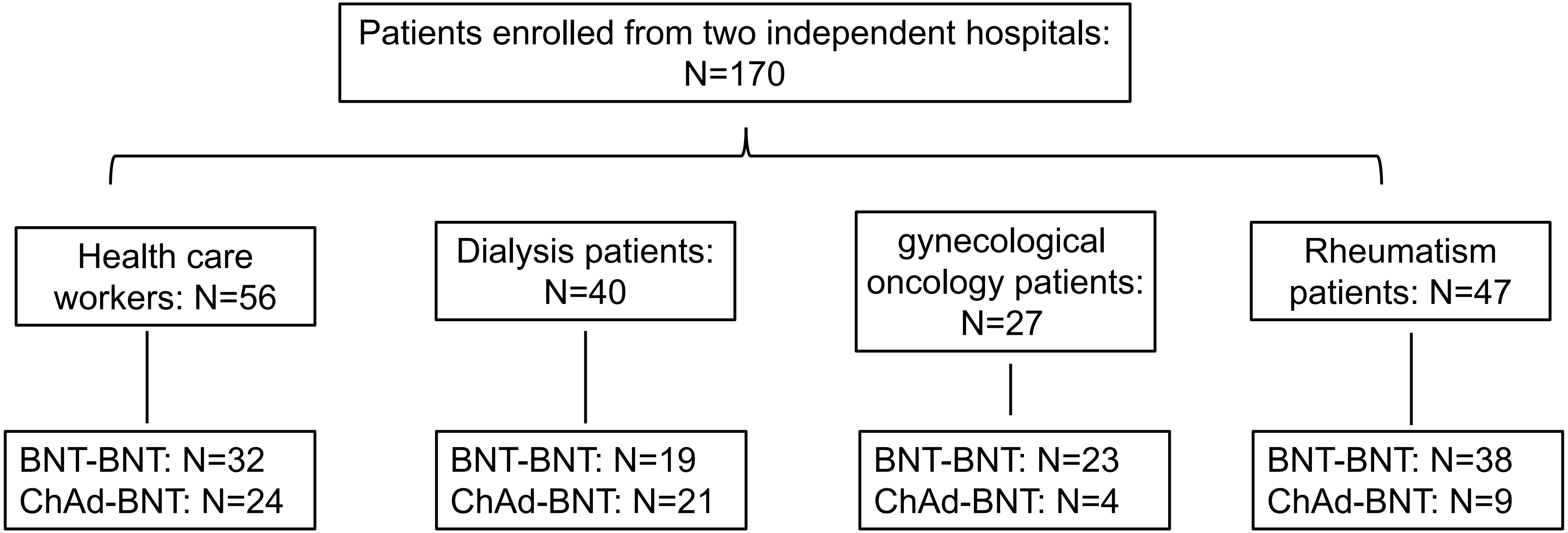

Supplementary Figure 1. This cohort consists of 170 participants from two independent hospitals, including 56 healthcare workers as healthy controls, of which 32 participants received the homologous regimen (BNT-BNT); 40 dialysis patients, of which 19 participants received the homologous regimen (BNT-BNT); 27 gynecological oncology patients, of which 23 participants received the homologous regimen (BNT-BNT), and 47 rheumatic patients, of which 38 participants received the homologous regimen (BNT-BNT).
